# Infantile pulmonary abscess due to *Mycobacterium abscessus* subsp. *massiliense* identified by integrated mNGS and targeted NGS: a rare case report

**DOI:** 10.3389/fped.2026.1828339

**Published:** 2026-06-12

**Authors:** Huanhuan Zhu, Yuting Lin, Hailing Liao, Xuming Li, Qingke Xie, Yinan Zheng

**Affiliations:** 1 Pediatric Intensive Care Unit, Guangdong Women and Children Hospital, Guangzhou, Guangdong, China; 2 Department of Scientific Affairs, Hugobiotech Co., Ltd., Beijing, China

**Keywords:** infant, mNGS, *Mycobacterium abscessus* subsp. *massiliense*, pulmonary abscess, targeted NGS

## Abstract

**Background:**

To describe a rare case of pulmonary infection caused by *Mycobacterium abscessus* in an infant and to evaluate the complementary diagnostic value of metagenomic next-generation sequencing (mNGS) and targeted next-generation sequencing (tNGS) in identifying non-tuberculous mycobacterial (NTM) infections when conventional testing is inconclusive.

**Case presentation:**

A 3-month-old male infant presented with a persistent cough and a right upper-lobe mass, initially suspected to be a congenital malformation or neoplasm. Following inconclusive routine examinations, mNGS was performed on bronchoalveolar lavage fluid (BALF). mNGS detected a single read of *M. abscessus* in BALF, providing an initial diagnostic clue. Subsequently, a tNGS assay was conducted on both BALF and resected lung tissue to achieve precise species identification. tNGS identified 13,272 reads of *M. abscessus subsp. massiliense* in BALF and 31,474 reads in lung tissue, confirming the pathogen and enabling precise molecular diagnosis. Histopathological examination revealed granulomatous inflammation with multinucleated giant cells, consistent with NTM infection. Guided by these results, the patient initially received azithromycin and was transferred to a specialized chest hospital, where a multidrug anti-NTM regimen was formulated, including azithromycin, imipenem-cilastatin, cefoxitin, and linezolid. After continued treatment at a local municipal hospital, respiratory symptoms resolved, inflammatory markers improved, follow-up CT showed progressive absorption of the right upper-lobe lesion with a small residual cavity, and the patient was discharged in stable condition without recurrent infections during available follow-up.

**Conclusion:**

This case highlights the diagnostic utility of integrating mNGS and tNGS for the accurate identification of rare NTM infections in infants, particularly when routine microbiological tests and imaging findings are inconclusive.

## Introduction

Non-tuberculous mycobacteria (NTM) are ubiquitous environmental pathogens whose clinical significance has received increasing attention in recent years (1). Among them, *Mycobacterium abscessus* is a rapidly growing species with intrinsic multidrug resistance, posing significant challenges for clinical diagnosis and treatment (2). Pediatric pulmonary infection due to *M. abscessus* is uncommon and typically occurs in patients with underlying conditions such as cystic fibrosis, chronic lung disease, or immunodeficiency (3–5). Pulmonary infection in immunocompetent infants without underlying disease is exceedingly rare. The clinical and radiologic features often mimic congenital pulmonary lesions or neoplasms, frequently resulting in misdiagnosis or delayed treatment.

The early recognition of NTM infections in infants is difficult due to nonspecific clinical manifestations, overlapping imaging findings, and the limited sensitivity of conventional microbiological tests. The advent of high-throughput sequencing technologies has transformed the diagnostic landscape. Metagenomic next-generation sequencing (mNGS) enables unbiased, culture-independent detection of multiple pathogens and has shown promise in identifying slow-growing or rare organisms, including NTM (6, 7). However, the diagnostic performance of mNGS in NTM infections is frequently constrained by the inherently low bacillary load and the organism's thick, lipid-rich cell wall, which together impede efficient nucleic acid release and result in markedly low sequencing reads (8). Furthermore, mNGS often fails to achieve reliable species or subspecies-level identification or to generate resistance-related data that are essential for guiding clinical management. Targeted next-generation sequencing (tNGS) overcomes these limitations by selectively amplifying high-copy or species-specific genomic regions of the *Mycobacterium tuberculosis* complex (MTBC) and NTM (9). This targeted enrichment substantially increases sensitivity in paucibacillary specimens and enables precise subspecies resolution, including differentiation of *M. abscessus* subspecies. This distinction is clinically vital, as it predicts macrolide susceptibility based on subspecies-dependent variations in *erm*(41) gene functionality (10, 11). Previous studies have shown that combining mNGS with tNGS can significantly enhance diagnostic accuracy for rare infections characterized by low pathogen abundance or inconclusive initial testing (12). Pulmonary infection caused by *M. abscessus* has rarely been reported in immunocompetent infants, and previous cases relied exclusively on conventional microbiological methods. To our knowledge, this case represents the first instance in which subspecies-level identification of *M. abscessus* was achieved directly from bronchoalveolar lavage fluid (BALF) and lung tissue using tNGS.

Here, we describe a rare case of pulmonary infection caused by *M. abscessus* subsp. *massiliense* in an infant who initially presented with a pulmonary mass suggestive of a congenital tumor. Initial BALF mNGS detected only a single *M. abscessus* read, whereas subsequent tNGS identified abundant *M. abscessus* subsp. *massiliense* sequences in both BALF and resected lung tissue, establishing the etiological diagnosis. The infant improved after expert-directed anti-NTM therapy, with gradual clinical, laboratory, and radiographic recovery and no recurrent infections during available follow-up. This case highlights the complementary value of mNGS and mycobacteria-focused tNGS in evaluating atypical pulmonary lesions caused by NTM in infants.

## Case description

**Figure 1:**
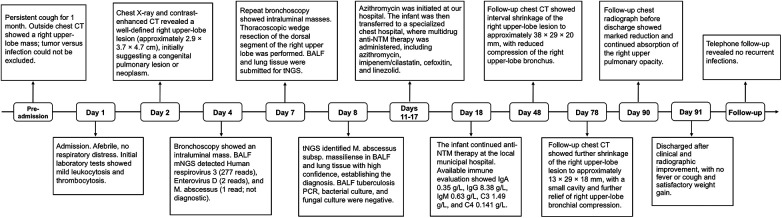
Clinical timeline of the patient from pre-admission to follow-up. CT, computed tomography; BALF, bronchoalveolar lavage fluid; mNGS, metagenomic next-generation sequencing; tNGS, targeted next-generation sequencing; PCR, polymerase chain reaction; NTM, nontuberculous mycobacteria; Ig, immunoglobulin; C, complement.

A male infant, aged 3 months and 26 days, was admitted with a one-month history of persistent cough. Twelve days prior to admission, chest computed tomography (CT) performed at an outside hospital revealed a right upper-lobe mass suspicious for a neoplastic lesion. The infant was delivered by cesarean section at 32 + 2 weeks of gestation to a previously healthy G5P4 mother with an unremarkable pregnancy history and a birth weight of 1.8 kg. There was no history of birth asphyxia or resuscitation; the amniotic fluid was clear, and no abnormalities of the umbilical cord or placenta were documented. There was no known history of tuberculosis or other infectious disease exposure. The infant had not received Bacillus Calmette–Guérin (BCG) vaccination before disease onset because vaccination had initially been deferred due to prematurity and low birth weight and was later postponed because of persistent cough lasting more than one month. No detailed environmental exposure mapping or environmental sampling was available. The infant had no known history of immunodeficiency; physical growth and neuropsychological development were comparable to those of age-matched infants. The parents were non-consanguineous. A timeline with relevant data and treatment from the patient in the course was shown in Figure 1.

**Figure 2:**
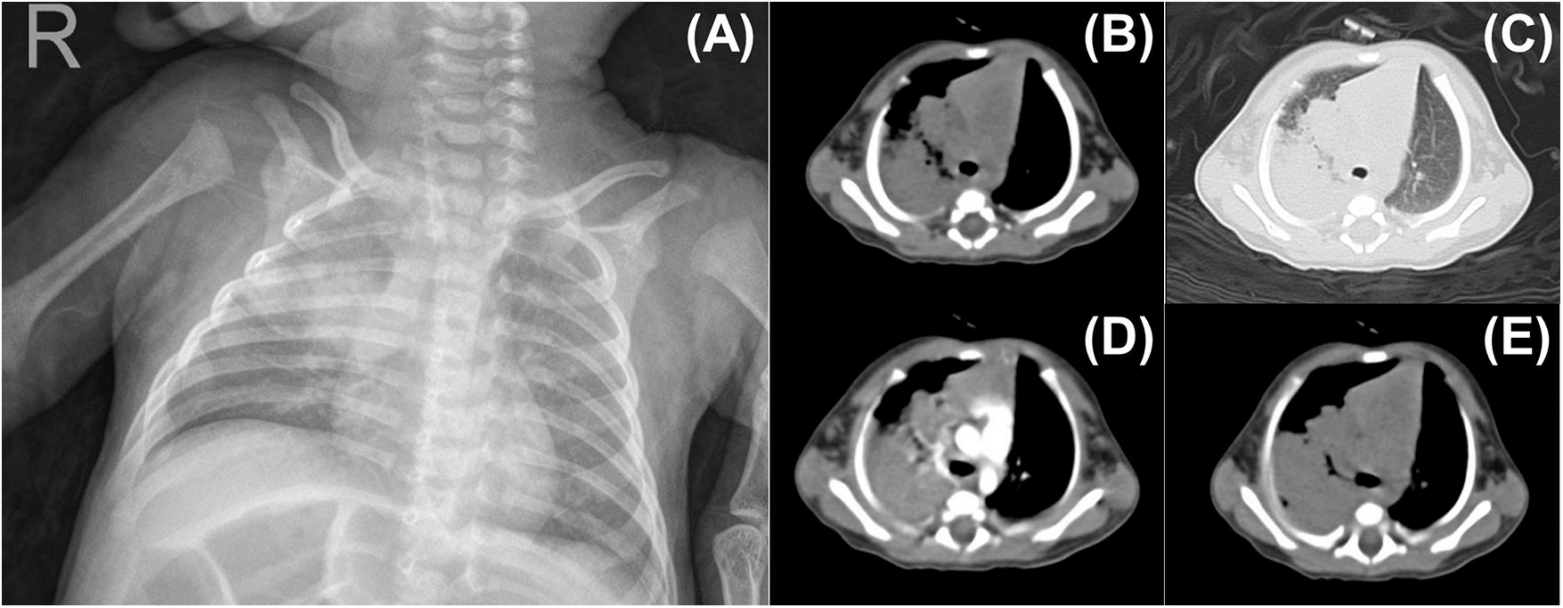
Chest radiograph and lung CT scan on admission. **(A)** The chest x-ray shows an abnormal opacity in the right upper lobe. **(B–E)** Lung CT on Day 2 demonstrates a heterogeneous mass in the right upper lobe measuring approximately 2.9 × 3.7 × 4.7 cm, with marked enhancement on contrast imaging.

**Figure 3:**
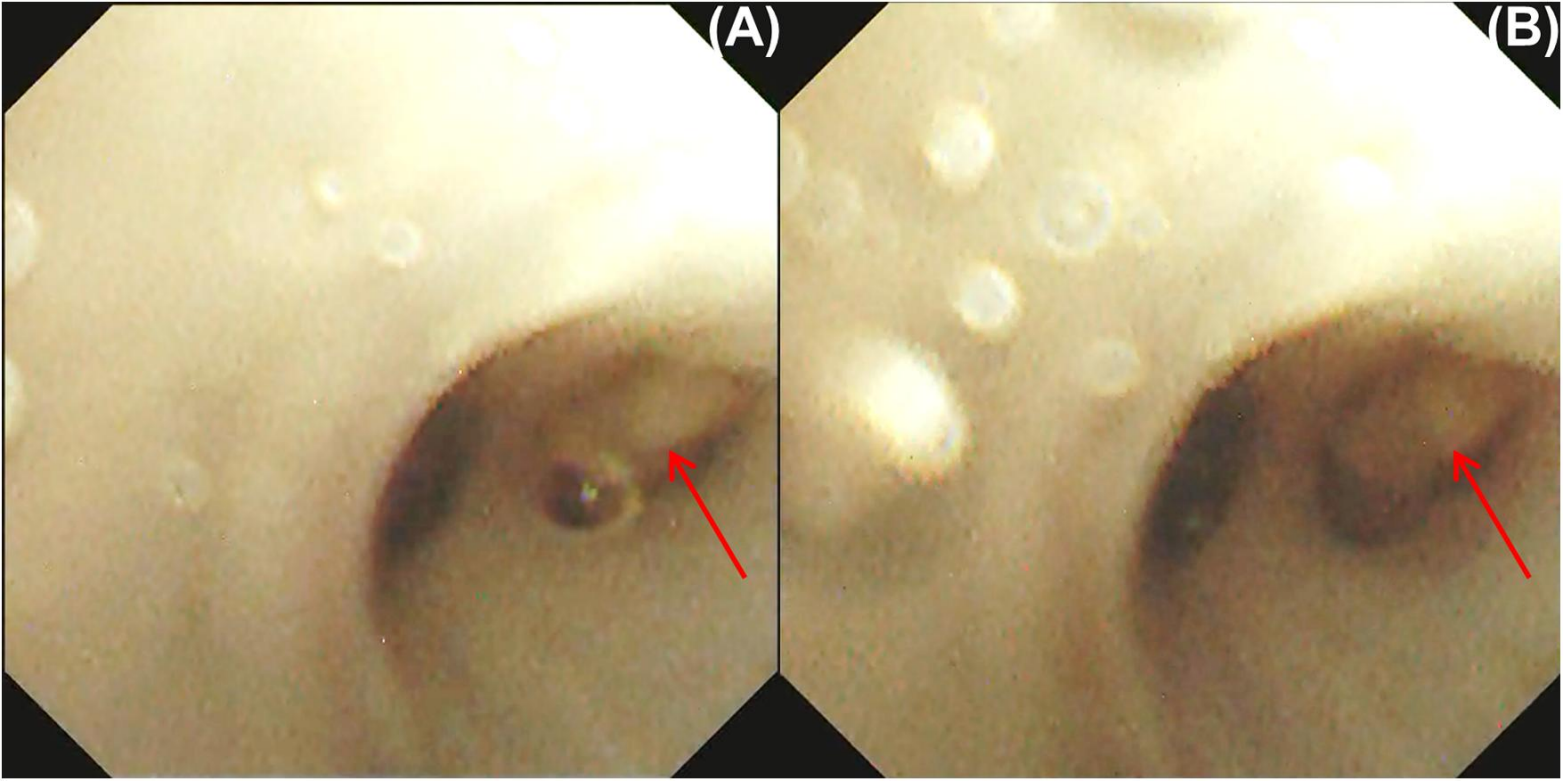
Bronchoscopy findings. **(A,B)** Bronchoscopy revealed intraluminal masses in both the apical and posterior segments of the right upper lobe. The lesions protruded into the airway lumen with irregular surfaces, resulting in luminal narrowing.

**Figure 4:**
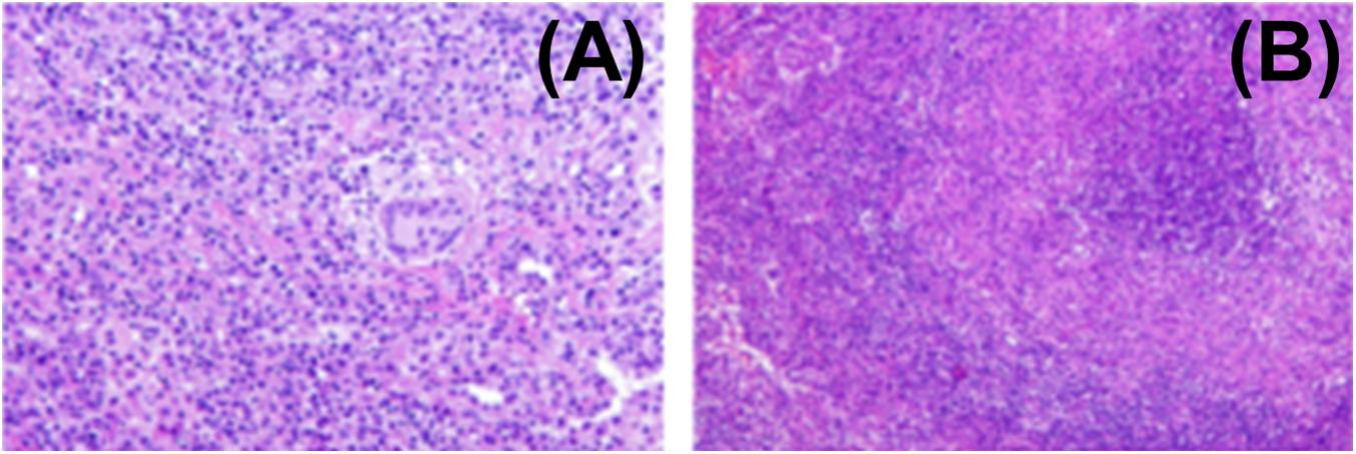
Histopathology of the lung tissue. **(A,B)** The lung tissue shows dense infiltration of inflammatory cells and histiocytes.

On Day 1, The infant was afebrile, exhibited cough without respiratory distress, and had no remarkable findings on physical examination. Laboratory results showed: White blood cell count (WBC): 15.88 × 10^9^/L, Neutrophil count (NEUT): 5.57 × 10^9^/L, Lymphocyte count (LYMPH): 8.32 × 10^9^/L, Hemoglobin (Hb): 108 g/L, Platelet count (PLT): 510 × 10^9^/L, C-reactive protein (CRP): 5.88 mg/L, Liver and renal function, coagulation parameters, and electrolytes were normal. On Day 2, the initial chest x-ray revealed an abnormal, dense opacity occupying the right upper lobe (Figure 2A). Subsequent contrast-enhanced CT of the chest demonstrated a well-defined, lobulated lesion in the right upper lobe (2.9 × 3.7 × 4.7 cm) with marked contrast enhancement (Figures 2B–E), raising suspicion for congenital peribronchial myofibroblastic tumor, although an infectious etiology could not be excluded. On Day 4, the diagnostic workup was escalated to bronchoscopy, which revealed a mass lesion in the subsegmental lumen of the right upper lobe posterior segment (Figure 3). The mass was observed to protrude into the airway with an irregular surface, resulting in significant luminal narrowing. BALF sample was immediately submitted for mNGS analysis. The mNGS assay included both DNA and RNA metagenomic sequencing. After library quality control, sequencing was performed on the Illumina 550DX platform with single-end 75-bp reads. Detailed laboratory workflow, bioinformatic analysis, quality control procedures, and interpretation criteria are provided in the Supplementary Materials. BALF DNA mNGS detected *M. abscessus* with 1 specific read, medium confidence, and relative abundance 0.25%. No fungi, DNA viruses, parasites, or resistance genes were detected in the DNA panel. BALF RNA mNGS detected Human respirovirus 3 (277 specific reads, high confidence, relative abundance 96.98%) and Enterovirus D (2 specific reads, medium confidence, relative abundance 1.47%). Given that the viral findings were inconsistent with the patient's clinical symptoms and severe radiological presentation, and the single *M. abscessus* read was deemed insufficient to establish a definitive diagnosis, a Multidisciplinary Team (MDT) consultation was convened. The MDT concluded that the diagnosis remained indeterminate and recommended a two-part strategy: (1) Proceed with thoracoscopic wedge resection of the lesion-containing lung tissue for definitive histopathological examination. (2) Immediately submit a fresh BALF sample for tNGS (TB-Pro assay developed by HugoBiotech), specifically designed for MTBC and NTM, to maximize the chance of pathogen confirmation and high-resolution subtyping. On Day 7, repeat bronchoscopy confirmed the presence of pale and significantly edematous bronchial mucosa, leading to narrowed lumens in the right lung segments. Grayish-white, tenacious secretions were adherent to the mucosa, which were cleared via suction. During lavage, two intraluminal masses were identified protruding into the airways of the right upper lobe apical and posterior segments (Figure 3), causing localized luminal stenosis. The endoscopic diagnosis was bronchial mucositis and right upper lobe apical/posterior segmental masses (nature undetermined). Subsequently, the patient underwent wedge resection of the dorsal segment of the right upper lobe. This procedure was a partial resection rather than lobectomy or fluid aspiration, and the resected lesion-containing lung tissue was submitted for histopathological examination to clarify the nature of the mass-like lesion (Figure 4). Following the inconclusive initial mNGS result and the MDT recommendation, further high-resolution molecular assays were pursued. BALF and resected lung tissue specimens were subjected to tNGS for MTBC and NTM. Detailed tNGS workflow and interpretation criteria are provided in the Supplementary Materials. These assays provided the definitive diagnosis. Conventional microbiological workup was also performed: BALF tuberculosis PCR was negative, and BALF bacterial and fungal cultures were negative. Ziehl-Neelsen staining and interferon-gamma release assay were not performed. Phenotypic antimicrobial susceptibility testing was unavailable because no viable mycobacterial isolate was obtained. The concurrent 10-item PCR nucleic acid detection panel performed on the BALF revealed co-detection of several potential pathogens: Cytomegalovirus (DNA), *Mycoplasma pneumoniae* (DNA), and Parainfluenza Virus Type 3 (RNA). Crucially, the tNGS assay (TB-Pro), provided the conclusive etiology: 13,272 reads of *M. abscessus* subsp. *massiliense* were detected in the BALF sample with high confidence, and an overwhelming 31,474 reads of the same subspecies were detected in the resected lung tissue, also with high confidence (Table 1). The high-confidence, high-abundance tNGS results, which confirmed the subspecies-level pathogen identification, allowed for immediate clinical correlation and supersedence of the auxiliary PCR findings. The final clinical diagnoses were thus established as: right pulmonary abscess (*M. abscessus* infection) and right upper lobe consolidation. On Day 7, laboratory tests showed WBC 17.79 × 10^9^/L, NEUT 7.04 × 10^9^/L, LYMPH 8.42 × 10^9^/L, Hb 107 g/L, PLT 469 × 10^9^/L, and CRP 8.91 mg/L. On Day 8, laboratory investigations revealed: WBC 11.88 × 10^9^/L, NEUT 7.14 × 10^9^/L, LYMPH 3.55 × 10^9^/L, Hb 94 g/L, PLT 406 × 10^9^/L, CRP 7.52 mg/L. Integrating clinical presentation, imaging, histopathology, and molecular testing, diagnosis of pulmonary abscess caused by *M. abscessus* subsp. *massiliense* was established. After the diagnosis was established, azithromycin suspension was initiated at our hospital from Day 11 to Day 14 at 9 mg/kg/day once daily, and the infant was transferred to a specialized chest hospital on Day 14 for further management. At admission to the chest hospital, blood tests showed WBC 18.45 × 10^9^/L, NEUT 8.10 × 10^9^/L, neutrophils 43.9%, lymphocytes 44.2%, Hb 94 g/L, and PLT 451 × 10^9^/L; cardiac, hepatic, renal, coagulation, and electrolyte parameters were unremarkable. A multidrug anti-nontuberculous mycobacterial (anti-NTM) regimen was administered, consisting of azithromycin suspension 9 mg/kg/day once daily, imipenem-cilastatin 20 mg/kg every 12 h, cefoxitin sodium 50 mg/kg every 8 h, and linezolid 10 mg/kg every 12 h. After 3 days in the chest hospital, the patient was discharged at the family's request and transferred back to a local municipal hospital to continue the chest-hospital-directed anti-NTM regimen.

**Table 1:** Pathogenic microorganisms were detected in BALF and lung tissue of the patient by tNGS.

Sample type	Genus Name	reads	Species Name	Specific reads
BALF	*Mycobacteroides*	20,175	*Mycobacteroides abscessus* subsp. *massiliense*	13,272
Lung tissue	*Mycobacteroides*	44,557	*Mycobacteroides abscessus* subsp. *massiliense*	31,474

The infant was hospitalized at the local municipal hospital for 74 days to continue anti-NTM therapy. On admission to the local hospital, blood tests showed WBC 22.58 × 10^9^/L, NEUT 8.10 × 10^9^/L, lymphocytes 12.11 × 10^9^/L, Hb 94 g/L, and PLT 451 × 10^9^/L. Immune-related testing showed immunoglobulin A (IgA) 0.35 g/L, immunoglobulin G (IgG) 8.38 g/L, immunoglobulin M (IgM) 0.63 g/L, complement component 3 (C3) 1.49 g/L, and complement component 4 (C4) 0.141 g/L. T-, B-, and natural killer-cell subset analysis (TBNK), neutrophil function testing, human immunodeficiency virus (HIV) testing, and immunodeficiency-related genetic testing were not performed. During continued treatment, sequential follow-up imaging demonstrated a favorable therapeutic response. Chest CT on local-hospital Day 31, corresponding to overall Day 48 after the initial admission to our hospital, showed shrinkage of the right upper-lobe mass to approximately 38 × 29 × 20 mm, with reduced bronchial compression (Supplementary Figures 1A-C). Chest CT on local-hospital Day 61, corresponding to overall Day 78, showed further reduction of the lesion to approximately 13 × 29 × 18 mm, with a small cavity and further relief of bronchial compression (Supplementary Figures 1D-E). Chest radiograph before discharge on local-hospital Day 73, corresponding to overall Day 90, showed continued reduction of the right upper pulmonary opacity (Supplementary Figure 1F). Following resolution of fever and cough, satisfactory weight gain, and radiographic improvement, the infant was successfully discharged on overall Day 91. No recurrent infections were reported during subsequent telephone follow-up.

## Discussion

This case highlights the multifaceted challenges in diagnosing infant pulmonary infections caused by NTM and underscores the value of sequentially combining mNGS and tNGS to achieve rapid and accurate pathogen identification. Pulmonary disease caused by *M. abscessus* is generally rare in children and is particularly uncommon in previously healthy infants. A systematic review of pulmonary NTM infection in infants showed that available evidence remains limited and is mainly derived from case reports and small case series (13). Reported pediatric risk factors include cystic fibrosis, chronic or structural lung disease, bronchiectasis, immunodeficiency, and environmental exposure to water- or soil-related reservoirs (14). Pediatric *M. abscessus* pulmonary infection has also been reported mainly in children with underlying lung pathology, especially cystic fibrosis or chronic pulmonary disease (15). According to the current literature, only around five cases of *M. abscessus* pulmonary infection have been identified in immunocompetent infants without preexisting lung diseases. Most of these patients developed symptoms between 2 and 4 months of age, including *M. abscessus* lung disease in a 4-month-old female infant (16), extensive *M. abscessus* pneumonia in a 4-month-old female infant (17), *M. abscessus* pneumonia in a 2-month-old immunocompetent male infant (18), *M. abscessus* pneumonia in a 53-day-old immunocompetent infant (19), and a familial outbreak of *M. abscessus* infection in immunocompetent 3-month-old triplets (20). In the present case, prematurity and low birth weight may have contributed to immature airway and immune defenses; however, no known immunodeficiency, recurrent infection pattern, or clear environmental source was identified. Collectively, these reports indicate that *M. abscessus*, although typically considered an opportunistic pathogen, can also cause severe pulmonary disease in infants without recognized immunodeficiency.

The diagnosis of nontuberculous mycobacterial pulmonary disease should be based on an integrated assessment of clinical, radiological, and microbiological evidence. The 2020 ATS/ERS/ESCMID/IDSA guideline continues to recommend the clinical, radiographic, and microbiologic diagnostic criteria originally described in the 2007 ATS/IDSA statement for classifying patients as having NTM pulmonary disease (21). According to this diagnostic framework, compatible pulmonary or systemic symptoms, compatible radiographic abnormalities, exclusion of alternative diagnoses, and microbiological evidence, such as positive cultures from sputum, BALF, or lung tissue, should be considered together (22). In the present case, the infant had persistent cough, a mass-like lesion in the right upper lobe on chest CT, and histopathological evidence of granulomatous inflammation with multinucleated giant-cell reaction. Conventional bacterial and fungal cultures of BALF were negative, BALF tuberculosis PCR was negative, and tNGS did not detect *Mycobacterium tuberculosis* complex. Although mycobacterial culture was unavailable and the guideline criteria were developed mainly for culture-based diagnosis, concordant high-confidence detection of *M. abscessus* subsp. *massiliense* in both BALF and resected lung tissue by tNGS, together with compatible clinical, radiological, and pathological findings, strongly supported true NTM pulmonary disease rather than airway contamination. This case also illustrates the need to interpret molecular results within the broader clinical context, especially in infants from whom repeated high-quality sputum samples and culture confirmation are often difficult to obtain.

However, the clinical and imaging manifestations of *M. abscessus* lung disease in infants are often non-specific, making it easily mistaken for more common conditions. In previous cases, *M. abscessus* pneumonia was frequently misdiagnosed as bacterial pneumonia or refractory pneumonia of unknown etiology (17). Some children even present with multiple nodular or mass-like lesions on CT, which necessitate differentiation from congenital pulmonary cystic lesions and neoplastic lesions (16). Furthermore, due to the low bacterial load and the dense, tough cell wall of mycobacteria, conventional microbiological examinations (including acid-fast staining and culture) often yield negative results in children with *M. abscessus* infection (20). mNGS offers the unique advantage of unbiased and broad-spectrum pathogen detection, enabling the identification of bacteria, fungi, viruses, and parasites without pre-set clinical hypotheses (23). In this case, a solid mass with necrosis in the right upper lung lobe initially suggested a neoplastic lesion based on imaging findings. Prompt collection of bronchoalveolar lavage fluid (BALF) for mNGS testing detected *M. abscessus* (1 read), providing a crucial clue for NTM infection and preventing misdiagnosis and inappropriate tumor-directed treatment. This highlights that for unexplained pulmonary lesions in infants, particularly those with atypical imaging that complicates differentiation from non-infectious conditions like tumors, early BALF mNGS testing is crucial for clarifying the lesion's etiology and guiding subsequent diagnostic and therapeutic decisions. Although Human respirovirus 3, also known as human parainfluenza virus 3, and Enterovirus D were detected by RNA mNGS, these findings were not sufficient to explain the localized mass-like lesion and granulomatous pathology. Respiratory viral infections, including parainfluenza virus infection, may contribute to secondary infection by impairing mucociliary clearance, disrupting airway epithelial barrier integrity, and modulating local immune responses (24, 25). However, whether these viral infections predisposed the infant to *M. abscessus* pulmonary disease remains uncertain, and a causal relationship could not be established in this single case.

Nevertheless, although mNGS is an excellent broad-spectrum screening method, its sensitivity may be limited by host background noise and low pathogen biomass, sometimes resulting in extremely low read counts. In this case, only one sequence of *M. abscessus* was detected by initial BALF mNGS. Although this result was insufficient for a confirmed diagnosis, it provided a critical directional clue in the context of negative conventional tests and strong clinical suspicion of infection. This prompted further mycobacteria-focused tNGS for the detection of MTBC and NTM. The sequential approach proved successful, as subsequent tNGS detected abundant *M. abscessus* subsp. *massiliense* sequences in both BALF and resected lung tissue, confirming the pathogen and its subspecies. The validity of this combined strategy is further supported by existing literature. In a case of infantile disseminated Bacillus Calmette-Guerin disease combined with X-linked severe combined immunodeficiency (X-SCID), mNGS detected MTBC in both blood and cerebrospinal fluid, and subsequent tNGS completed the analysis of *Mycobacterium bovis* and its drug resistance genes, thereby clarifying the infection type and drug resistance characteristics (12). Another case of occult migratory MTBC related secondary organizing pneumonia showed that when mNGS was negative and pathogen abundance was extremely low, tNGS successfully detected *Mycobacterium tuberculosis* in lung tissue, confirming the pathogen (26). To our knowledge, this case represents the first reported instance of infantile pulmonary abscess caused by *M. abscessus* subsp. *massiliense* confirmed by tNGS. This finding not only emphasizes the advantages of tNGS in diagnosing rare or fastidious mycobacterial infections but also provides a vital clinical recommendation. For complex pediatric pulmonary lesions, particularly those presenting as masses or abscesses with conventional tests yielding negative results, the integration of mNGS and tNGS should be actively considered as the optimal diagnostic pathway to expedite pathogen identification and minimize the use of empirical anti-infective therapy.

Treatment of *M. abscessus* pulmonary disease is challenging and should ideally be individualized according to subspecies identification, disease severity, drug susceptibility testing when an isolate is available, and expert consultation. Current ATS/ERS/ESCMID/IDSA guidance emphasizes susceptibility-based treatment, particularly for macrolides and amikacin, and recommends multidrug therapy for *M. abscessus* pulmonary disease (22). In this case, the regimen formulated by the specialized chest hospital included azithromycin, imipenem-cilastatin, cefoxitin, and linezolid. Subsequent treatment was associated with clinical resolution, decreasing inflammatory markers, and progressive radiological absorption of the lesion.

Beyond diagnosis and treatment, this case also raises clinically relevant issues regarding host susceptibility and infection-control management. The patient had not received BCG vaccination before disease onset. BCG vaccination was initially deferred because of prematurity and low birth weight and was later not administered because of persistent cough for more than one month. Evidence regarding the protective effect of BCG against NTM disease is mixed and appears to be disease- and species-dependent. A systematic review suggested that BCG vaccination was associated with a reduced risk of childhood NTM lymphadenitis and short-term protection against Buruli ulcer (27), and experimental evidence suggests that BCG can induce cross-reactive immune responses against *M. avium* and *M. abscessus* (28). However, direct clinical evidence supporting protection against *M. abscessus* pulmonary disease remains limited. Therefore, the absence of BCG vaccination was considered a relevant clinical background rather than a proven risk factor in this case. From an infection-control perspective, distinguishing NTM from MTBC is also important. NTM differ substantially from MTBC because they are generally acquired from environmental reservoirs, including water, soil, dust, and moist built environments (29). In this case, MTBC was not detected by tNGS; therefore, routine chemoprophylaxis for household contacts was not indicated. Standard precautions and clinical follow-up were recommended. Enhanced infection-control measures may be considered in specific settings, such as suspected healthcare-associated transmission, exposure to contaminated water sources, or high-risk populations (21).

In conclusion, this case highlights the importance of including NTM, such as *M. abscessus*, in the early differential diagnosis for infants without known immunodeficiency presenting with unexplained pulmonary masses or abscess-like lesions. When conventional etiological examinations yield negative or limited results, the combined application of mNGS and tNGS can effectively compensate for these diagnostic shortcomings. tNGS offers distinct advantages, particularly when subspecies-level identification is necessary to guide antimicrobial regimen selection. In this case, the combined mNGS and tNGS approach enabled rapid confirmation of *M. abscessus* subsp. *massiliense* as the causative pathogen of infantile pulmonary abscess, providing a robust foundation for accurate clinical management.

## Limitations

This report has several limitations. Primarily, as a single case study, these findings regarding the integrated use of mNGS and tNGS may not be universally generalizable to all pediatric NTM infections. While this combined application was decisive in our diagnostic pathway, its cost-effectiveness and specific clinical indications require further validation in larger, prospective cohorts. Although the subsequent treatment regimen and short-term recovery course were obtained, longer clinical and radiological follow-up is still required because *M. abscessus* pulmonary disease often requires prolonged multidrug therapy and may relapse. In addition, the exact origin of the infection remains unidentified, as no environmental sampling or detailed exposure mapping was conducted to trace the pathogen's source. Immunological evaluation was incomplete: serum immunoglobulin and complement levels were available, but lymphocyte subset analysis, neutrophil function testing, HIV testing, and immunodeficiency-related genetic testing were not performed. The lack of mycobacterial culture and phenotypic antimicrobial susceptibility testing also limited culture-based confirmation and drug susceptibility-guided interpretation.

## Data Availability

The original contributions presented in the study are publicly available. This data can be found here: National Genomics Data Center (NGDC), accession number PRJCA066134.
