# Supplementary material for: Infantile pulmonary abscess due to Mycobacterium abscessus subsp. massiliense identified by integrated mNGS and targeted NGS: a rare case report
Source: Front Pediatr. 2026 Jun 12;14:1828339. doi: 10.3389/fped.2026.1828339 (PMC13306562; doi:10.3389/fped.2026.1828339)
Supplement: Supplementary file 1 [file Supplementaryfile1.docx]

Supplementary Material

**Next-generation sequencing and bioinformatic analysis**

Bronchoalveolar lavage fluid and resected lung tissue specimens were subjected to pathogenic next-generation sequencing assays, including metagenomic next-generation sequencing (mNGS) and targeted next-generation sequencing (tNGS) for Mycobacterium tuberculosis complex and nontuberculous mycobacteria. Clinical samples for mNGS were shipped to HugoBiotech Co., Ltd. (Beijing, China).

For DNA mNGS, DNA was extracted using the Nucleic Acid Extraction Reagent Kit (YGZZ014-48, HugoBiotech, Beijing, China), and sequencing libraries were constructed using the Universal Sequencing Reaction Kit (YGZZ005, HugoBiotech, Beijing, China). For RNA mNGS, RNA was extracted using the Nucleic Acid Extraction Reagent Kit (YGZZ009, HugoBiotech, Beijing, China), and libraries were constructed using the Universal Sequencing Reaction Kit (YGZZ010, HugoBiotech, Beijing, China). DNA mNGS was used for the detection of bacteria, fungi, DNA viruses, and parasites, whereas RNA mNGS was used for the detection of RNA viruses. For each experimental batch, a no-template control was included using sterile water as the initial input. The concentration and integrity of the sequencing libraries were measured using Qubit 4.0 (Thermo Fisher Scientific, USA) and the Agilent 2100 Bioanalyzer (Agilent Technologies, USA). After library quality control, sequencing was performed on the Illumina 550DX platform (Illumina, Inc., USA) with single-end 75-bp reads. After obtaining the raw data, bcl2fastq (v2.20.0.422) with default parameters was employed to convert the “bcl” files into fastq format and to split data of each sample in a batch. Then, fastp (v0.24.0) (1), with its default parameters, was used to filter out adapters, low-quality sequences and sequences with high percentage of “N”, thus generating high quality sequences with a minimum length greater than 50 bp. Next, BWA (0.7.15) (2) of “mem” mode was used to align the sequences to the human genome (GRCh38.101), thereby removing host-derived sequences. The remaining reads were then aligned to a local database using BWA (0.7.15), which was compiled from NCBI Refseq and GenBank databases. Subsequently, microbial identification was carried out based on the alignment results. The number of unique aligned reads and RPM (reads per million) for each identified microorganism were calculated.

Positive mNGS results were defined according to the following criteria: For the detected bacteria (*Mycobacterium* excluded), fungi (*Cryptococcus* excluded), and parasites, the positive criteria for the mNGS result were set as follows: (1) genome coverage of the unique reads mapped to this microorganism ranked top10 of the same kind of microbes and the microorganism was not detected in the NTC; or (2) RPM-r (RPM_sample_/RPM_NTC)_ was > 10 (RPM_NTC_ ≠ 0). For viruses, *Mycobacterium tuberculosis*, and *Cryptococcus*, a positive mNGS result was considered when it was not detected in NTC and at least 1 unique read was mapped to species or when RPM-r was > 5 (RPM_NTC_ ≠ 0).

For tNGS detection, 200 μL of sample was used for DNA and RNA extraction with the Nucleic Acid Extraction Reagent Kit (YGZZ017, HugoBiotech, Beijing, China). RNA was reverse-transcribed using the 1st Strand cDNA Synthesis Kit (YG-048, HugoBiotech, Beijing, China). Target enrichment was performed using the Multi-Pathogen Detection Reagent Kit (YG-032, HugoBiotech, Beijing, China), which was developed based on the MultiPrime primer design platform (3). Library construction was performed using the Universal Sequencing Reaction Kit (YGZZ018, HugoBiotech, Beijing, China). Library concentration and integrity were assessed using Qubit 4.0 and the Agilent 2100 Bioanalyzer. After quality control, sequencing was performed on the Illumina 550DX platform with single-end 75-bp reads.

For tNGS result report, microbial identification was subsequently carried out based on the RPM, RPM-r and detected primer ratio (detected primer pair number/designed primer pair number). If the RPM of targeted pathogens in tested samples is greater than 100, RPM_-r_ was > 5 (RPM_NTC_ ≠ 0), and the detected primer ratio higher than 0.1, the result is considered positive. For tNGS result reporting, microbial identification was performed based on three key metrics: reads per million (RPM), RPM ratio (RPM-r), and detected primer ratio (number of detected primer pairs / total designed primer pairs). A positive result was defined when the following criteria were met: RPM of targeted pathogens in test samples exceeded 100, RPM-r was > 5 (RPM_NTC_ ≠ 0); and detected primer ratio was higher than preset requirements.

**Supplementary Figure 1.** Follow-up chest CT and chest radiograph during anti-nontuberculous mycobacterial (NTM) treatment. (A-C) Chest CT on local-hospital Day 31, corresponding to overall Day 48, shows interval shrinkage of the right upper-lobe mass-like lesion to approximately 38 × 29 × 20 mm, with heterogeneous enhancement and reduced bronchial compression. (D-E) Chest CT on local-hospital Day 61, corresponding to overall Day 78, shows further reduction of the lesion to approximately 13 × 29 × 18 mm, with a small cavity and focal non-enhancing areas suggestive of necrotic change; compression of the right upper-lobe bronchus is further relieved. (F) Chest radiograph before discharge on local-hospital Day 73, corresponding to overall Day 90, shows decreased right upper pulmonary opacity compared with previous imaging.


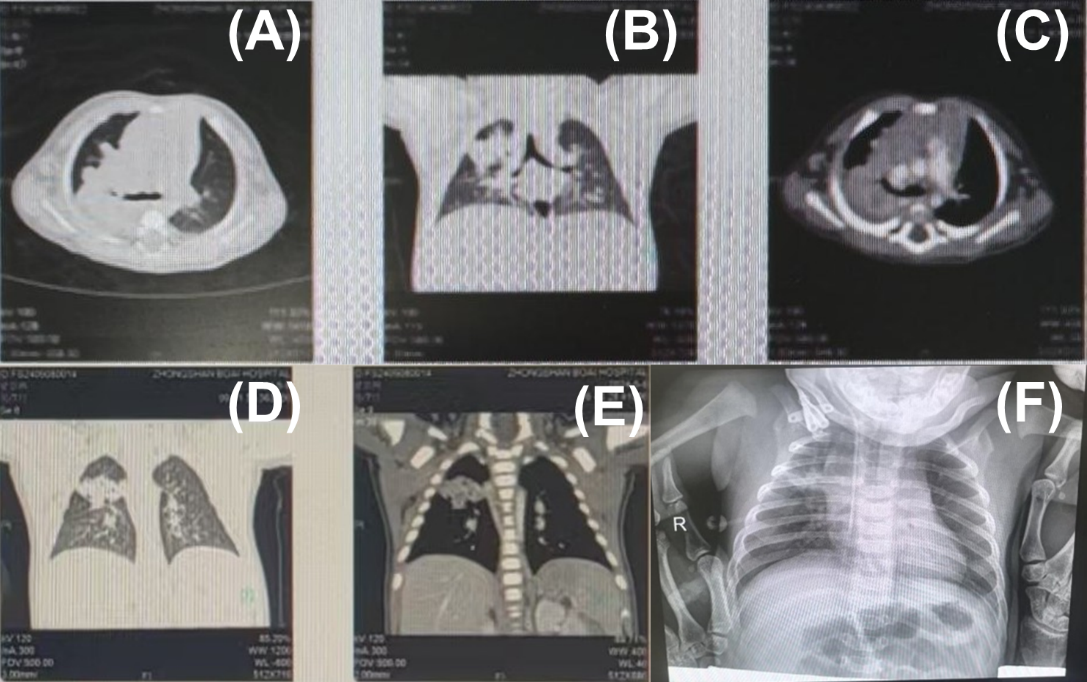


**Reference：**

1. Chen S, Zhou Y, Chen Y, Gu J. fastp: an ultra-fast all-in-one FASTQ preprocessor. *Bioinformatics* (2018) 34:i884–i890. doi: 10.1093/bioinformatics/bty560

2. Li H, Durbin R. Fast and accurate long-read alignment with burrows–wheeler transform. *Bioinformatics* (2010) 26:589–595. doi: 10.1093/bioinformatics/btp698

3. Xia H, Zhang Z, Luo C, Wei K, Li X, Mu X, Duan M, Zhu C, Jin L, He X, et al. MultiPrime: a reliable and efficient tool for targeted next-generation sequencing. *iMeta* (2023) 2:e143. doi: 10.1002/imt2.143
